# Liver transplantation for chronic hepatitis C virus infection in the United States 2002–2014: An analysis of the UNOS/OPTN registry

**DOI:** 10.1371/journal.pone.0186898

**Published:** 2017-10-31

**Authors:** Georg Dultz, Barry I. Graubard, Paul Martin, Martin-Walter Welker, Johannes Vermehren, Stefan Zeuzem, Katherine A. McGlynn, Tania M. Welzel

**Affiliations:** 1 University Hospital Frankfurt, Frankfurt am Main, Germany; 2 Biostatistics Branch, Division of Cancer Epidemiology and Genetics, National Cancer Institute, Bethesda, MD, United States of America; 3 Hepatology Division, University of Miami, Miami, FL, United States of America; 4 HREB, Division of Cancer Epidemiology and Genetics, National Cancer Institute, Bethesda, MD, United States of America; Istituto Mediterraneo per i Trapianti e Terapie ad Alta Specializzazione, ITALY

## Abstract

Chronic hepatitis C virus (HCV) infection is a leading cause for orthotopic liver transplantation (OLT) in the U.S. We investigated characteristics of HCV-infected patients registered for OLT, and explored factors associated with mortality. Data were obtained from the United Network for Organ Sharing and Organ Procurement and Transplantation network (UNOS/OPTN) registry. Analyses included 41,157 HCV-mono-infected patients ≥18 years of age listed for cadaveric OLT between February 2002 and June 2014. Characteristics associated with pre- and post-transplant survival and time trends over the study period were determined by logistic and Cox proportional hazard regression analyses and Poisson regressions. Most patients were white (69.1%) and male (70.8%). At waitlist registration, mean age was 54.6 years and mean MELD was 16. HCC was recorded in 26.9% of the records. A total of 51.2% of the patients received an OLT, 21.0% died or were too sick; 15.6% were delisted and 10.4% were still waiting. Factors associated with increased waitlist mortality were older age, female gender, blood type 0, diabetes, no HCC and transplant region (p<0.001). OLT recipient characteristics associated with increased risk for post OLT mortality were female gender, age, diabetes, race (p<0,0001), and allocation MELD (p = 0.005). Donor characteristics associated with waitlist mortality included age, ethnicity (p<0.0001) and diabetes (p<0.03). Waitlist registrations and OLTs for HCC significantly increased from 14.4% to 37.3% and 27.8% to 38.5%, respectively (p<0.0001). Pre- and post-transplant survival depended on a variety of patient-, donor-, and allocation- characteristics of which most remain relevant in the DAA-era. Still, intensified HCV screening strategies and timely and effective treatment of HCV are highly relevant to reduce the burden of HCV-related OLTs in the U.S.

## Introduction

Chronic infection with hepatitis C virus (HCV) is a major risk factor for liver cirrhosis and hepatocellular carcinoma (HCC) [1]. To date, HCV-related complications are a leading cause for orthotopic liver transplantation (OLT) in the U.S [2]. Epidemiological studies indicate that 2.7 million persons or 1.0% of the U.S. population are chronically infected with HCV. Highest HCV prevalences are reported in persons born between 1945 and 1965 [3]. The relatively high prevalence of HCV infection in this “baby boomer” birth cohort may be attributable to high rates of new infections among young adults in the 1970s and 1980s mainly due to contaminated blood products, drug abuse and high risk sexual behavior [3]. According to current CDC estimates, approximately three-fourths of all HCV infected persons in the U.S. belong to the 1945 to 1965 birth cohort [4]. Duration of HCV infection represents, together with some viral and host factors, a major risk factor for progressive liver disease [5, 6]. Due to an aging population, underdiagnosis of the HCV infection and poor sustained virologic response rates (SVR) to historic pegylated-interferon-based therapies, the prevalence of HCV related complications continued to increase over the last decades [7].At the current time, HCV-associated morbidity and mortality is higher than for any other infectious disease in the United States [8]. In addition, despite recently implemented changes in screening strategies, approximately 50% of HCV infected persons are unaware of their infection and are at risk of developing advanced liver disease [9]. Despite some decrease in HCV-related end-stage liver disease due to the impact of highly effective direct-acting antivirals (DAAs) HCV and HCV-related HCC is likely to prevail as an important cause for liver transplantation in the foreseeable future. That is why the characterization of HCV infected patients listed for OLT and the factors associated with their survival a topic of interest [9, 10]. In this study, we investigated epidemiological characteristics of HCV mono-infected patients registered for liver transplantation at UNOS/OPTN and explored factors associated with waitlist and post-transplant mortality.

## Patients and methods

### Study population

The data for this analysis were obtained from the United Network for Organ Sharing and Organ Procurement and Transplantation network (UNOS/OPTN) registry, a secure transplant information database contains all data in the U.S. on the waiting list, organ donation and matching, and transplantation [11, 12]. This study includes all patients aged 18 years or older with a primary or secondary diagnosis of chronic HCV mono-infection registered at UNOS/OPTN for cadaveric OLT between 27/FEB/2002 (data of MELD score implementation) and 30/JUN/2014. We excluded patients with a diagnosis of acute HCV infection, alcoholic liver disease, viral co-infections, living donor transplantations, and patients with status 1A (high urgency). Assessment of the cause of liver disease and other demographic and clinical variables followed the UNOS/OPTN coding of variables (primary and secondary diagnosis). A flow chart of how the study cohort was derived from the entire registry is shown in **S1 Fig**. Briefly, a total of 137,589 patients with a diagnosis of chronic HCV infection were registered for OLT at UNOS/OPTN during the time period of interest. Excluded from the current analysis were 9,735 patients <18 years of age; 2,384 patients who received a living donor transplant; 5,670 patients who were registered with high urgency status 1A; and 78,643 patients with documented viral HIV or HBV co-infection or documented alcoholic liver disease.

### Statistical analysis

Descriptive comparisons were made between the HCV patientswith and without HCC for demographic and clinical variables that were used in the logistic and Cox proportional hazard regression analyses. Unconditional logistic regression was used to compare the 21,064 patients who received a transplant with the 8,634 patients who died or became too sick to be transplanted. The covariates used in the logistic regression analysis were selected using backwards stepwise logistic regression where covariates were chosen if the two-sided p-value for prediction of death or being too ill was smaller than 0.10. The primary outcome studied in this paper was overall survival after transplant of HCV patients on the waitlist. Cox proportional hazard regression was used to identify predictors of survival while on the waitlist. The time metric for the Cox regression survival analyses was follow-up time from transplant to death or end of the follow-up period (30/JUN/2014), whichever came first, where patients who were alive at end of the follow-up period were censored observations. Forward stepwise Cox regression analyses were conducted for selecting the independent predictors with two-sided p-values that were less than 0.05 based on likelihood ratio testing. Using results from the Cox regression analyses, direct adjusted survival curves were plotted to display the relationships between specific groups of patients. Between 2003–2013, Poisson regression models were used to test for trends in the annual rates of liver transplant waitlist registrations and annual rates of transplants by including year as a continuous covariate and an offset for the annual US population size of individuals 18 years and older. Between 2003–2013 logistic regression was used to test for trends in the annual proportion of patients with HCV among those patients on the waitlist, and in the annual proportion of patients with HCC among those patients with HCV. All data analyses were performed using SAS (version 9.4) and all p-values were two-sided without correction for multiple comparisons.

## Results

### Patient population

The demographic and clinical characteristics of 41,157 HCV mono-infected patients listed for OLT during the study period are displayed in **Table 1.**

**Table 1:** Demographic and clinical characteristics of HCV-infected patients register for OLT in the United States between 27-FEB-2002 and 30-JUN-2014.

Patient characteristics	Overall population (N = 41,157)	Patients without HCC (N = 30,082)	Patients withHCC (N = 11,075)
Male sex, N(%)	29,141 (70.8)	20,518 (68.2)	8,623 (77.9)
Age, mean ± SD			
Male	54.3 ± 6.9	53.2 ± 7.0	56.9 ± 6.2
Female	55.3 ± 7.8	54.5 ± 7.8	58.4 ± 7.0
Race/Ethnicity, N(%)			
White	28,447 (69.1)	21,220 (70.5)	7,227 (65.3)
Black	4,616 (11.2)	3,215 (10.7)	1,401 (12.7)
Hispanic	6,365 (15.5)	4,663 (15.5)	1,702 (15.4)
Asian	1,271 (3.1)	662 (2.2)	609 (5.5)
Other	458 (1.1)	322 (1.1)	136 (1.2)
Lab-MELD, N(%)			
MELD <10	7,640 (18.6)	3,509 (11.7)	4,131 (37.3)
MELD 10–14	14,271 (34.7)	10,009 (33.3)	4,262 (38.5)
MELD 15–19	9,450 (23.0)	7,813 (26.0)	1,637 (14.8)
MELD 20–24	4,629 (11.2)	4,053 (13.5)	576 (5.2)
MELD 25–29	2,014 (4.9)	1,822 (6.1)	192 (1.7)
MELD 30–34	1,325 (3.2)	1,184 (3.9)	141 (1.3)
MELD 35+	1,777 (4.3)	1,650 (5.5)	127 (1.1)
Missing	51 (0.1)	42 (0.1)	9 (0.1)
Lab-MELD, mean± SD	16.0 ± 7.9	17.3 ± 8.2	12.3 ± 5.9
Allocation MELD, N(%)			
MELD <10	7,547 (18.3)	3,449 (11.5)	4,098 (37.0)
MELD 10–14	14,076 (34.2)	9,837 (32.7)	4,239 (38.3)
MELD 15–19	9,330 (22.7)	7,706 (25.6)	1,624 (14.7)
MELD 20–24	4,586 (11.1)	4,015 (13.3)	571 (5.2)
MELD 25–29	1,982 (4.8)	1,792 (6.0)	190 (1.7)
MELD 30–34	1,315 (3.2)	1,174 (3.9)	141 (1.3)
MELD 35+	1,751 (4.3)	1,626 (5.4)	125 (1.1)
TemporarilyInactive	570 (1.4)	483 (1.6)	87 (0.8)
BMI, mean ± SD			
Male	28.6 ± 5.0	28.7 ± 5.1	28.4 ± 4.7
Female	28.8 ± 6.0	28.8 ± 6.0	28.4 ± 5.7
Laboratory values, mean ± SD			
Albumin	3.0 ±0.7	2.9 ±0.7	3.18 ±0.7
Bilirubin	4.2 ±7.0	4.8 ±7.7	2.39 ±4.1
IRN	1.5 ±0.8	1.6 ±0.9	1.33 ±0.4
Serum creatinine	1.3 ±1.2	1.4 ±1.3	1.03 ±0.8
ABO blood type, N(%)			
O	19,218 (46.7)	14,124 (47.0)	5,094 (46.0)
A	15,256 (37.1)	11,138 (37.0)	4,118 (37.2)
AB	1,663 (4.0)	1,217 (4.0)	446 (4.0)
B	5,020 (12.2)	3,603 (12.0)	1,417 (12.8)
Encephalopathy, N(%)			
Absent	16,341 (39.7)	9,830 (32.7)	6,511 (58.8)
Present	24,759 (60.2)	20,206 (67.2)	4,553 (41.1)
Unknown	57 (0.1)	46 (0.2)	11 (0.1)
Ascites, N(%)			
Absent	10,821 (26.3)	5,637 (18.7)	5,184 (46.8)
Present	30,279 (73.6)	24,399 (81.1)	5,880 (53.1)
Unknown	57 (0.1)	46 (0.2)	11 (0.1)
Diabetes, N(%)			
No	31,220 (75.9)	22,897 (76.1)	8,323 (75.2)
Yes	9,393 (22.8)	6,720 (22.3)	2,673 (24.1)
Unknown	544 (1.3)	465 (1.5)	79 (0.7)
Region, N (%)*			
1	1,813 (4.4)	1,200 (4.0)	613 (5.5)
2	5,513 (13.4)	4,072 (13.5)	1,441 (13.0)
3	5,218 (12.7)	3,980 (13.2)	1,238 (11.2)
4	5,105 (12.4)	3,912 (13.0)	1,193 (10.8)
5	8,061 (19.6)	5,741 (19.1)	2,320 (20.9)
6	1,391 (3.4)	927 (3.1)	464 (4.2)
7	2,428 (5.9)	1,851 (6.2)	577 (5.2)
8	2,322 (5.6)	1,673 (5.6)	649 (5.9)
9	3,343 (8.1)	2,296 (7.6)	1,047 (9.5)
10	2,641 (6.4)	1,894 (6.3)	747 (6.7)
11	3,322 (8.1)	2,536 (8.4)	786 (7.1)

The database showed only very few missing values: 0.1% for encephalopathy, ascites and labMELD and 1.3% for diabetes at baseline.

*States and areas in regions of OPTN: 1 Connecticut, Maine, Massachusetts, New Hampshire, Rhode Island, Vermont; 2 Delaware, District of Columbia,Maryland, New Jersey, Pennsylvania, Northern Virginia, West Virginia; 3 Alabama, Arkansas, Florida, Georgia, Louisiana, Mississippi, Puerto Rico 4 Oklahoma, Texas;5 Arizona, California, Nevada, New Mexico, Utah; 6 Alaska, Hawaii, Idaho, Montana, Oregon, Washington; 7 Illinois, Minnesota, North Dakota, South Dakota, Wisconsin;8 Colorado, Iowa, Kansas, Missouri, Nebraska, Wyoming; 9 New York; 10 Indiana, Michigan, Ohio; 11 Kentucky, North Carolina, South Carolina, Tennessee, Virginia.

The majority of the patients were male (70.8%). The racial/ethnic composition of the population was 69.1% white, 15.5% Hispanic, 11.2% black and 3.1% Asian. The mean age of the OLT candidates was 54.6 years, and similar in men (54.3 yrs.) and women (55.3 yrs). A lab- and allocation MELD ≥10 was reported for 81.3% and 80.3% of the patients, respectively. The mean lab-MELD score was 16.0 ±8 at the time of waitlist registration. Blood types 0 (46.7%) and A (37.1%) were the most common.

With regard to clinical characteristics, signs of hepatic decompensation such as hepatic encephalopathy (60.2%) and ascites (73.6%) were documented in most patients. A history of diabetes was recorded in 22.8% of the patients. Over half of the HCV-related registrations for liver transplantation (58.1%) were recorded for regions 2 to 5. A total of 11,075 (26.9%) of the patients had HCV-related HCC.

Demographic and baseline characteristics of the patients listed for OLT with and without a diagnosis of HCC are summarized in **Table 1**. Compared to the patients without HCC, patients with HCC were older but had less advanced liver disease and its complications such as encephalopathy or ascites. In addition, a greater proportion of Asians and blacks were listed with HCV-related HCC. There were no major differences, however, regarding blood type, prevalence of diabetes and transplant region.

### OLT waitlist disposition and characteristics associated with waitlist mortality

**Fig 1:**
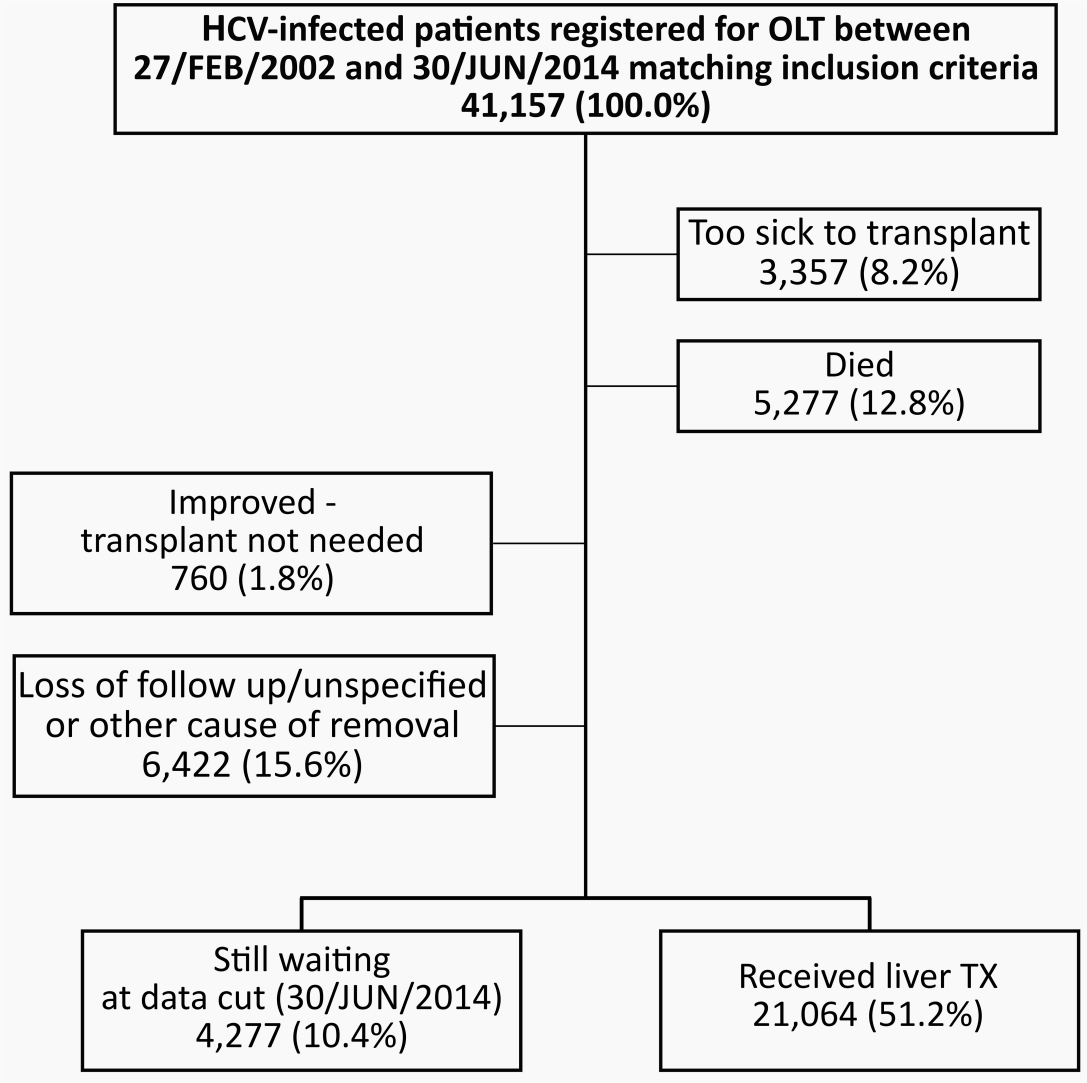
Patient disposition of hepatitis C infetected patients registered for liver transplantation between 27/FEB/2002 and 30/JUN/2014. Over half of the waitlist registrants received a liver transplantation while 20% where too sick to transplant or died on the waitlist.

Of the 41,157 patients included in the study, 51.2% received an OLT. Reasons for waitlist removal included: too sick to be transplanted (8.2%), death (12.8%), patient condition improved (1.8%), and loss-to-follow up/other cause/not specified (15.6%). A total of 4,277 of 41,157 patients (10.4%) were still on the waitlist at the end date of the study (**Fig 1)**.

To identify factors associated with waitlist mortality, we compared patients that either died or were removed for being too sick to transplant, patients that received an OLT (**Table 2**).

**Table 2:** Characteristics associated with waitlist mortality by univariate and multivariate logistic regression analysis*.

			Univariate analysis		Multivariate analysis	
	OLT recipients	Died on waitlistor removed	Odds ratio (95% CI)	p-value	Odds ratio (95% CI)	p-value
Gender, N (%)				<0.0001		<0.0001
Male	15,560 (73.9)	5,756 (66.7)	Ref.		Ref.	
Female	5,504 (26.1)	2,878 (33.3)	1.414 (1.34–1.49)		1.261 (1.19–1.34)	
Age, mean ± SD	54.3 ± 7.1	55.2 ± 7.1	1.017 (1.01–1.02)	<0.0001	1.032 (1.03–1.03)	<0.0001
Race/Ethnicity, N (%)				<0.0001		0.68
White	14,639 (69.5)	5,809 (67.3)	Ref.		Ref.	
Black	2,569 (12.2)	960 (11.1)	0.94 (0.87–1.02)		0.941 (0.86–1.03)	
Hispanic	2,977 (14.1)	1,493 (17.3)	1.26 (1.18–1.35)		0.975 (0.90–1.05)	
Asian	643 (3.1)	265 (3.1)	1.04 (0.90–1.20)		1.022 (0.87–1.20)	
Other	236 (1.1)	107 (1.2)	1.14 (0.91–1.44)		1.026 (0.80–1.32)	
HCV related HCC, N (%)				<0.0001		<0.0001
No	13,259 (63.9)	7,332 (84.9)	Ref.		Ref.	
Yes	7,805 (37.1)	1,301 (15.1)	0.30 (0.28–0.32)		0.248 (0.23–0.27)	
ABO blood type, N (%)				<0.0001		<0.0001
0	9,404 (44.6)	4,302 (49.8)	Ref.		Ref.	
A	7,773 (36.9)	3,224 (37.3)	0.91 (0.86–0.96)		0.92 (0.86–0.97)	
AB	1,109 (5.3)	200 (2.3)	0.39 (0.34–0.46)		0.35 (0.29–0.41)	
B	2,778 (13.2)	908 (10.5)	0.71 (0.66–0.78)		0.66 (0.60–0.72)	
Diabetes, N (%)				<0.0001		<0.0001
No	16,219 (78.0)	6,317 (74.3)	Ref.		Ref.	
Yes	4,563 (22.0)	2,184 (25.7)	1.23 (1.16–1.30)		1.21 (1.13–1.28)	
Region, N (%)**				<0.0001		<0.0001
1	763 (3.6)	453 (5.3)	Ref.		Ref.	
2	2,737 (13.0)	1,264 (14.6)	0.78 (0.68–0.89)		0.68 (0.59–0.79)	
3	3,631 (17.2)	703 (8.1)	0.33 (0.28–0.38)		0.26 (0.22–0.30)	
4	2,152 (10.2)	1,071 (12.4)	0.84 (0.73–0.96)		0.73 (0.63–0.85)	
5	3,166 (15.0)	2,052 (23.8)	1.09 (0.96–1.24)		1.002 (0.87–1.15)	
6	810 (3.9)	242 (2.8)	0.50 (0.42–0.61)		0.474 (0.39–0.58)	
7	1,291 (6.1)	502 (5.8)	0.66 (0.56–0.77)		0.531 (0.45–0.63)	
8	1,344 (6.4)	427 (5.0)	0.54 (0.46–0.63)		0.476 (0.40–0.56)	
9	1,351 (6.4)	981 (11.4)	1.22 (1.06–1.41)		1.135 (0.98–1.32)	
10	1,710 (8.1)	431 (5.0)	0.43 (0.36–0.50)		0.371 (0.32–0.44)	
11	2,109 (10.0)	508 (5.9)	0.41 (0.35–0.47)		0.340 (0.21–0.40)	

* Adjusted for co-Variates as displayed in Table 1.

**States and areas in regions of OPTN: 1 Connecticut, Maine, Massachusetts, New Hampshire, Rhode Island, Vermont; 2 Delaware, District of Columbia, Maryland, New Jersey, Pennsylvania, Northern Virginia, West Virginia; 3 Alabama, Arkansas, Florida, Georgia, Louisiana, Mississippi, Puerto Rico 4 Oklahoma, Texas; 5 Arizona, California, Nevada, New Mexico, Utah; 6 Alaska, Hawaii, Idaho, Montana, Oregon, Washington; 7 Illinois, Minnesota, North Dakota, South Dakota, Wisconsin; 8 Colorado, Iowa, Kansas, Missouri, Nebraska, Wyoming; 9 New York; 10 Indiana, Michigan, Ohio; 11 Kentucky, North Carolina, South Carolina, Tennessee, Virginia.

In the univariate analysis, female gender (OR 1.41; 95%CI 1.34–1.49), older age (1.02; 95%CI 1.01–1.02), Hispanic ethnicity (1.26, 95%CI 1.18–1.35) and presence of diabetes (1.23; 95%CI 1.16–1.30) were significantly associated with increased waitlist mortality. Presence of HCC (OR 0.30, 95%CI 0.28–0.32) and of blood groups other than 0 were associated with decreased waitlist mortality. Significant differences were also observed for OPTN regions **(Table 2).**

In the multivariate analysis, all of the above variables except Hispanic ethnicity were significantly associated with waitlist mortality. In particular, female gender (OR 1.26, 95%CI 1.19–1.33), age (per year) (OR 1.03, 95%CI 1.03–1.04), presence of diabetes (OR 1.21, 95%CI 1.13–1.28) and blood group 0 were independently associated with increased waitlist mortality. Observed differences by OPTN region remained significant in the multivariate analysis.

### Factors associated with overall post-OLT survival

**Table 3** shows the results of the analysis that examined factors associated with overall survival among the OLT recipients.

**Table 3:** Recipient, donor and other characteristics associated with overall survival of HCV-infected OLT recipients.

		Univariate analysis		Multivariate analysis*	
		Hazard ratio (95% CI)	p-value	Hazard ratio (95% CI)	p-value
**Recipient Characteristics**					
Gender			<0.0001		<0.0001
Male	15,048 (73.8)	Ref.		Ref.	
Female	5,337 (26.2)	1.18 (1.11–1.25)		1.17 (1.10–1.25)	
Age	55.0±7.1	1.02 (1.01–1.02)	<0.0001	1.02 (1.01–1.02)	<0.0001
Race/Ethnicity			<0.0001		<0.0001
White	14,158 (69.5)	Ref.		Ref.	
Black	2,512 (12.3)	1.44 (1.34–1.55)		1.31 (1.21–1.42)	
Hispanic	2,862 (14.0)	0.95 (0.88–1.03)		0.89 (0.81–0.97)	
Asian	624 (3.1)	0.92 (0.78–1.08)		0.90 (0.75–1.07)	
Other	229 (1.1)	0.88 (0.67–1.16)		0.88 (0.67–1.17)	
Lab-MELD			<0.0001		0.33
MELD <10	2,660 (13.1)	1.05 (0.95–1.16)		1.02 (0.92–1.14)	
MELD 10–14	4,377 (21.5)	Ref.		Ref.	
MELD 15–19	4,332 (21.3)	0.95 (0.88–1.04)		1.03 (0.93–1.13)	
MELD 20–24	3,264 (16.0)	1.14 (1.04–1.24)		1.11 (0.98–1.25)	
MELD 25–29	1,976 (9.7)	1.21 (1.09–1.34)		1.19 (1.03–1.39)	
MELD 30–34	1,510 (7.4)	1.31 (1.17–1.46)		1.23 (1.03–1.47)	
MELD 35+	2,266 (11.1)	1.42 (1.29–1.57)		1.23 (1.00–1.52)	
Allocation MELD			<0.0001		0.005
MELD <10	163 (0.8)	1.17 (0.88–1.55)		1.20 (0.90–1.64)	
MELD 10–14	683 (3.4)	Ref.		Ref.	
MELD 15–19	2,736 (13.4)	0.84 (0.72–0.98)		0.85 (0.71–1.00)	
MELD 20–24	6,532 (32.0)	1.10 (0.95–1.26)		1.04 (0.87–1.25)	
MELD 25–29	5,150 (25.3)	1.09 (0.95–1.26)		1.02 (0.84–1.23)	
MELD 30–34	2,418 (11.9)	1.21 (1.03–1.41)		1.10 (0.89–1.36)	
MELD 35+	2,701 (13.3)	1.40 (1.20–1.63)		1.19 (0.93–1.51)	
Albumin	3.0±0.7	0.96 (0.92–0.99)	0.02	0.94 (0.90–0.98)	0.007
Exception case			0.15		0.19
No	12,142 (59.6)	Ref.		Ref.	
Yes	8,243 (40.4)	0.96 (0.91–1.02)		1.08 (0.96–1.20)	
ABO blood type			0.63		0.32
0	9,091 (44.6)	Ref.		Ref.	
A	7,514 (36.9)	0.99 (0.93–1.05)		1.04 (0.98–1.11)	
AB	1,074 (5.3)	0.94 (0.83–1.06)		1.03 (0.91–1.18)	
B	2,706 (13.3)	0.96 (0.88–1.05)		0.96 (0.88–1.05)	
Encephalopathy			0.02		0.27
Absent	7,217 (35.4)	Ref.		Ref.	
Present	13,168 (64.6)	1.07 (1.01–1.13)		1.04 (0.97–1.12)	
Ascites			0.17		0.82
Absent	4,657 (22.9)	Ref.		Ref.	
Present	15,728 (77.1)	1.05 (0.98–1.12)		1.01 (0.93–1.10)	
Diabetes			<0.0001		<0.0001
No	15,913 (78.1)	Ref.		Ref.	
Yes	4,471 (21.9)	1.33 (1.25–1.41)		1.25 (1.17–1.33)	
Dialysis preTX			<0.0001		<0.0001
No	18,464 (90.6)	Ref.		Ref.	
Yes	1,921 (9.4)	1.52 (1.40–1.66)		1.36 (1.23–1.51)	
**Donor Characteristics**					
Gender			<0.001		0.75
Male	12,291 (60.3)	Ref.		Ref.	
Female	8,094 (39.7)	1.10 (1.04–1.16)		0.99 (0.94–1.05)	
Age	40.4±15.9	1.02 (1.02–1.02)	<0.0001	1.02 (1.02–1.02)	<0.0001
Ethnicity			<0.0001		<0.0001
White	13,567 (66.6)	Ref.		Ref.	
Black	3,446 (16.9)	0.96 (0.89–1.03)		0.92 (0.85–0.99)	
Hispanic	2,623 (12.9)	1.17 (1.08–1.26)		1.26 (1.15–1.36)	
Asian	473 (2.3)	1.44 (1.23–1.69)		1.39 (1.18–1.64)	
Other	276 (1.4)	1.14 (0.91–1.43)		1.33 (1.05–1.68)	
Diabetes			<0.0001		0.03
No	18,329 (89.9)	Ref.		Ref.	
Yes	2,056 (10.1)	1.37 (1.26–1.49)		1.10 (1.01–1.20)	
HCV-antibody status			0.69		0.48
Negative	18,872 (92,6)	Ref.		Ref.	
Positive	1,472 (7.2)	1.033 (0.93–1.15)		1.06 (0.95–1.19)	
Not reported	41 (0.2)	0.84 (0.48–1.48)		0.85 (0.47–1.55)	
**Independent Characteristics**					
Cold Ischemic Time	7.0±3.4	1.01 (1.01–1.02)	<0.01	1.01 (1.00–1.02)	<0.01
Transplant procedure			0.16		0.63
Whole liver	20,137 (98.8)	Ref.		Ref.	
Split/partial liver	248 (1.2)	0.83 (0.64–1.08)		1.07 (0.81–1.41)	
Region, N (%)*			<0.0001		<0.0001
1	749 (3.7)	Ref.		Ref.	
2	2,645 (13.0)	1.04 (0.90–1.21)		1.02 (0.87–1.20)	
3	3,537 (17.4)	0.85 (0.73–0.99)		0.91 (0.77–1.06)	
4	2,121 (10.4)	0.84 (0.72–0.98)		0.89 (0.75–1.05)	
5	3,030 (14.9)	0.82 (0.70–0.96)		0.75 (0.64–0.88)	
6	785 (3.9)	0.70 (0.57–0.85)		0.76 (0.62–0.94)	
7	1,240 (6.1)	0.98 (0.82–1.16)		0.92 (0.77–1.10)	
8	1,322 (6.5)	0.79 (0.66–0.94)		0.87 (0.72–1.04)	
9	1,295 (6.4)	1.12 (0.95–1.33)		0.96 (0.81–1.14)	
10	1,660 (8.1)	0.89 (0.76–1.05)		0.97 (0.82–1.15)	
11	2,001 (9.8)	0.87 (0.74–1.02)		0.95 (0.80–1.13)	
Transplant year			0.05		0.006
2002	594 (2.9)	0.96 (0.83–1.12)		1.022 (0.870–1.201)	
2003	1,263 (6.2)	0.99 (0.88–1.11)		0.991 (0.874–1.124)	
2004	1,546 (7.6)	Ref.		Ref.	
2005	1,613 (7.9)	1.01 (0.91–1.14)		0.99 (0.88–1.12)	
2006	1,763 (8.7)	0.98 (0.87–1.10)		0.94 (0.83–1.06)	
2007	1,753 (8.6)	1.00 (0.89–1.12)		1.00 (0.88–1.13)	
2008	1,687 (8.3)	1.16 (1.03–1.30)		1.12 (0.99–1.26)	
2009	1,853 (9.1)	1.06 (0.94–1.19)		0.97 (0.86–1.10)	
2010	1,824 (9.0)	1.00 (0.88–1.14)		0.91 (0.80–1.04)	
2011	1,837 (9.0)	0.91 (0.80–1.04)		0.85 (0.73–0.98)	
2012	1,911 (9.4)	0.92 (0.79–1.07)		0.84 (0.72–0.98)	
2013	1,832 (9.0)	0.91 (0.76–1.09)		0.83 (0.69–1.00)	
2014	909 (4.5)	1.21 (0.83–1.78)		1.08 (0.74–1.57)	

Values in column 2 are absolute N (%) or mean±SD

*States and areas in regions of OPTN: 1 Connecticut, Maine, Massachusetts, New Hampshire, Rhode Island, Vermont; 2 Delaware, District of Columbia, Maryland, New Jersey, Pennsylvania, Northern Virginia, West Virginia; 3 Alabama, Arkansas, Florida, Georgia, Louisiana, Mississippi, Puerto Rico 4 Oklahoma, Texas; 5 Arizona, California, Nevada, New Mexico, Utah; 6 Alaska, Hawaii, Idaho, Montana, Oregon, Washington; 7 Illinois, Minnesota, North Dakota, South Dakota, Wisconsin; 8 Colorado, Iowa, Kansas, Missouri, Nebraska, Wyoming; 9 New York; 10 Indiana, Michigan, Ohio; 11 Kentucky, North Carolina, South Carolina, Tennessee, Virginia. Multivariate analyses adjust for all other covariates.

In the univariate analysis, several recipient factors were associated with decreased post-transplant survival including female gender (HR 1.18; 95%CI 1.11–1.25), age (per year) (HR 1.02, 95%CI 1.01–1.02), black race (HR 1.44, 95%CI 1.34–1.55), lab-MELD and allocation MELD scores in categories between 20 and 35+, hepatic encephalopathy (HR 1.07, 95%CI 1.01–1.13), diabetes (HR 1.33, 95%CI 1.25–1.41) and pretreatment dialysis (HR 1.52, 95%CI 1.40–1.65).

Donor characteristics associated with decreased post-transplant survival were female gender (HR 1.10, 95%CI 1.04–1.16), older age (HR 1.02, 95%CI 1.01–1.02), Asian race (HR 1.44, 95%CI 1.23–1.69), Hispanic ethnicity (HR 1.17, 95%CI 1.08–1.26), and diabetes (HR 1.37, 95%CI 1.26–1.49).

In addition to recipient and donor factors, increase in cold ischemia time was associated with significantly decreased survival (HR 1.01, 95%CI 1.01–1.02) (**Table 3).**

No association with survival was observed for blood type, transplant procedure (partial/split liver vs. whole liver) and HCV antibody status of the donor.

Most of the variables that were associated with survival in the univariate analysis remained significant when adjusted for all other covariates. Among recipient characteristics, female gender (HR 1.17, 95%CI 1.10–1.25), older age (HR 1.02, 95%CI 1.01–1.02), black race (HR 1.31, 95%CI 1.21–1.42), allocation MELD scores in categories between 20 and 35+, diabetes (HR 1.25, 95%CI 1.17–1.33) and pretreatment dialysis (HR 1.36, 95%CI 1.23–1.51) were associated with significantly reduced post-transplant survival, whereas patients with Hispanic ethnicity had a better survival compared to white patients (HR 0.89, 95%CI 0.81–0.97).

Among donor characteristics, older age (HR 1.02, 95%CI 1.02–1.02), diabetes (HR 1.10, 95%CI 1.01–1.20, p<0.0001), Hispanic ethnicity (HR 1.22, 95% CI 1.15–1.36) and Asian race (HR 1.34, 95%CI 1.18–1.64) were associated with decreased survival, as was cold ischemia time (HR 1.01, 95%CI 1.00–1.02). In contrast, black race of the donor was associated with significantly better survival (HR 0.92, 95%CI 0.85–0.99). In the adjusted survival analyses significant differences were observed between transplant regions and across transplant years.

### Time trends in waitlist registrations and transplantations 2002–2014

To understand the changes in HCV-related disease burden in the United States 2002–2014 the number of HCV-related waitlist registrations and transplantations were analyzed. The time trends are summarized in **Table 4**.

**Table 4:** Wait list registrations and transplantations (liver) per year.

Year	All adult OLT waitlist registrations, N*	Waitlist registrations with HCV, N (% of all registrations)	Waitlist registrations with HCV-related HCC, N (% of all HCV)	All adult liver transplants, N	Transplants for HCV, N (% of all TX)	Transplants for HCV-related HCC, N (% of all TX for HCV)
2002**	7,106	2,268 (31.9)	283 (12.5)	1,768	616 (34.8)	213 (34.6)
2003	9,225	2,988 (32.4)	429 (14.4)	3,674	1,300 (35.4)	362 (27.8)
2004	9,840	3,250 (33.0)	471 (14.5)	4,522	1,606 (35.5)	421 (26.2)
2005	10,110	3,147 (31.1)	588 (18.7)	4,936	1,685 (34.1)	456 (27.1)
2006	10,186	3,185 (31.3)	686 (21.5)	5,365	1,802 (33.6)	483 (26.8)
2007	10,259	3,260 (31.8)	867 (26.6)	5,272	1,803 (34.2)	572 (31.7)
2008	10,344	3,404 (32.9)	895 (26.3)	5,172	1,859 (35.9)	627 (33.7)
2009	10,471	3,487 (33.3)	1,054 (30.2)	5,261	1,915 (36.4)	670 (35.0)
2010	11,225	3,624 (32.3)	1,196 (33.0)	5,218	1,863 (35.7)	677 (36.3)
2011	11,206	3,692 (32.9)	1,264 (34.2)	5,359	1,872 (34.9)	728 (38.9)
2012	10,923	3,651 (33.4)	1,370 (37.5)	5,314	1,947 (36.6)	733 (37.6)
2013	11,276	3,500 (31.0)	1,307 (37.3)	5,489	1,860 (33.9)	716 (38.5)
2014**	5,683	1,701 (29.9)	665 (39.1)	2,652	936 (35.3)	352 (37.6)
Σ	127,854	41,157 (32.2)	11,075 (26.9)	60,002	21,064 (35.1)	7,010 (33.3)
Trend 2003–2013***	p<0.0001	p = 0.44	p<0.0001	p<0.0001	p = 0.35	p<0.0001

* All indications

** Incomplete years were excluded from the analyses.

*** Regression models were used to test for trends in the annual rates of liver transplant registrations and transplants (column 2 and 5; Poisson regression) as well as the proportion of patients with HCV and HCC (column 4, 5, 6, and 7; logistic regression) between 2003 and 2013.

While the rate of waitlist registrations (the number of registrations provided in Table 4 divided by US Census population size) as well as the rate of adult liver transplantations (number of transplantations provided in Table 4 divided by US Census population size) increased significantly between 2003 and 2013 [rate ratio of registrations: RR (ratio of the rate for a year to the previous year) = 1.0062, 95%CI 1.00–1.01, p<0.0001; rate ratio of transplants: RR = 1.01, 95%CI 1.01–1.02, p-value<0.0001]. The proportion of HCV patients among all registrations [OR (odds ratio for a change per year) = 1.00, 95%CI 1.00–1.01, p = 0.44] and the proportion of HCV among all transplants (OR = 1.00, 95%CI 1.00–1.01), p = 0.35) remained stable over the study period. In contrast, the proportion of patients with HCC among the HCV registration cohort increased over the study period as reflected by the steady increase from 14.4% in 2003 to 37.3% in 2013 (OR = 1.14, 95%CI 1.13–1.15), p<0.0001). In parallel, the proportion of HCC-related transplants among the HCV transplant cohort increased significantly from 27.7% to 38.5% (OR = 1.07, 95%CI 1.06, 1.08), p<0.0001).

To investigate and compare possible changes in the characteristics of HCV-related waitlist registrants between 2002 and 2014 the study period was divided into three periods (2002–2005, 2006–2009, 2010–2014). Results are summarized in **S1 Table.** The age of patients increased, as did the proportion of black patients (p<0.0001) and the proportion of patients with diabetes (p<0.0001). A significant increase in registrations of patients with HCC was observed over the time periods (p<0.0001). Conversely, significant decreases were evident in the percentage of patients with encephalopathy (p<0.0001) and ascites (p<0.0001). Small statistically significant differences of lab values (albumin, bilirubin, INR and creatinine) and BMI were also observed over time. The baseline characteristics of registrations for the subgroup of patients with HCC in the three time periods are summarized in **S2 Table**. Changes between the time periods followed similar trends as did the whole study cohort with an increase in the number patients with diabetes, black race and older ages, and a decrease in the number of patients with encephalopathy or ascites.

**Fig 2:**
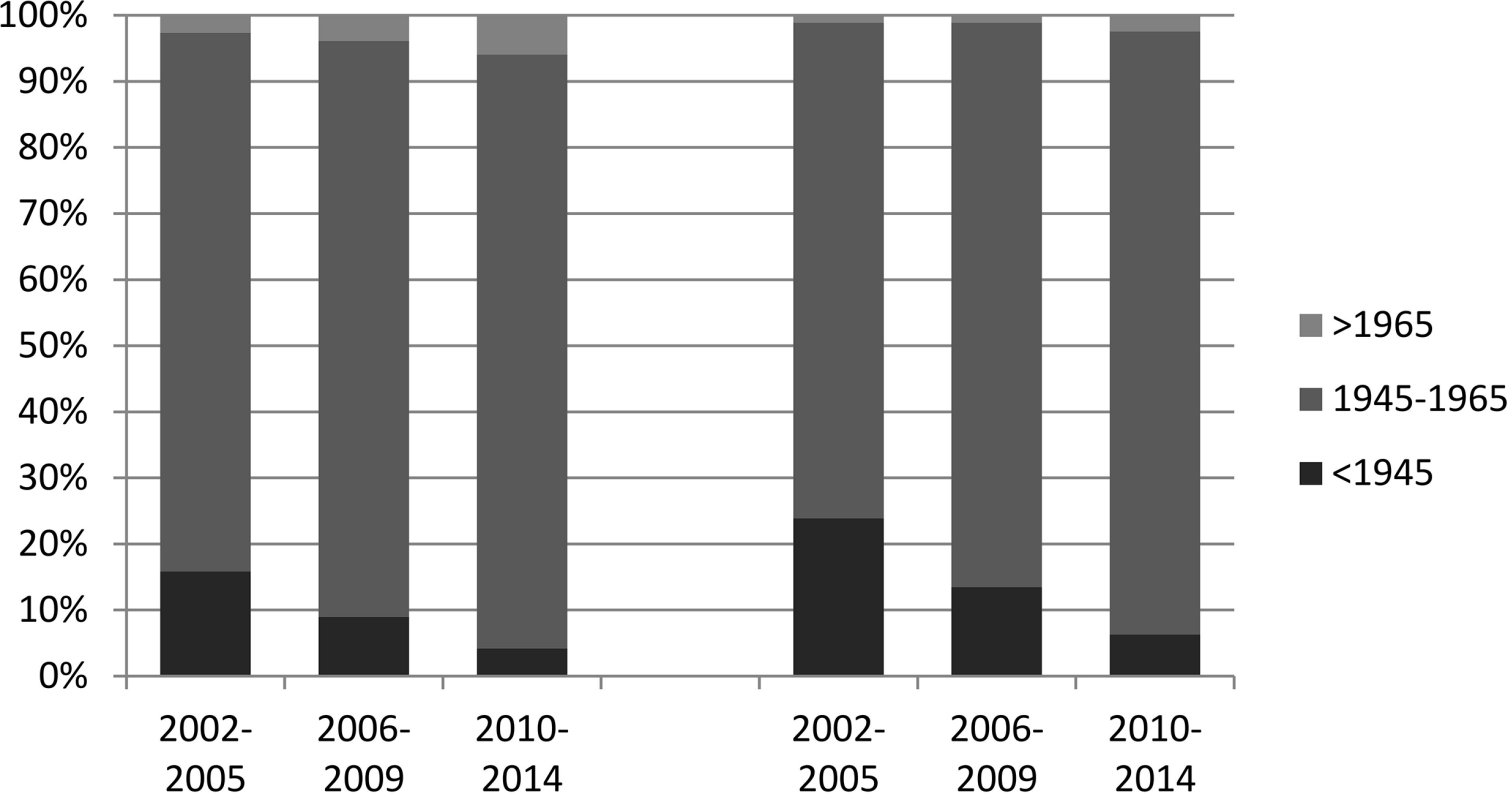
Proportion of baby boomers in the cohort of waitlist registrations overall and in the subgroup with HCC. An increasing number of registrations with HCV and HCV-related HCC belonged to the baby boomer cohort.

Patients born between 1945 and 1965 (“baby boomers”) accounted for 86.7% of all waitlist registrations from 2002 to 2014. Among the patients with HCC, a similar proportion of 86.8% (9,616 of 11,975) were baby boomers. The baby boomer proportion of all waitlist registrations increased gradually from 81.6% in 2002–2005 to 87.1% in 2006–009 and 90.0% in 2010–2014.The increase was even more pronounced in the subgroup of patients that registered with HCC (75.0%– 85.4% - 91.3% baby boomers). The data on baby boomers are summarized in **Table 5** and are illustrated in **Fig 2**.

**Table 5:** Birth cohort distribution of waitlist registrations.

	2002–2014		2002–2005		2006–2009		2010–2014	
	Total, N (%)	HCC, N (%)	Total, N (%)	HCC, N (%)	Total, N (%)	HCC, N (%)	Total, N (%)	HCC, N (%)
<1945	3,718 (9.0)	1,265 (11.4)	1,847(15.8)	424 (23.9)	1,195(9.0)	474(13.5)	676(4.2)	367(6.3)
1945–1965	35,666 (86.7)	9,616(86.8)	9,506(81.6)	1,328(75.0)	11,615(87.1)	2,990(85.4)	14,545(90.0)	5,298(91.3)
>1965	1,773(4.3)	194(1.8)	300(2.6)	19(1.1)	526(3.9)	38(1.1)	947(5.9)	137 (2.4)

## Discussion

In this large population based study we investigated OLT waitlist registrations and post-transplant outcomes among HCV-infected patients in the United States during the MELD era. The study underlines that chronic HCV infection is a major cause of end-state liver disease in the United States accounting for a third of waitlist registrations (41,157 of 127,854; 32.2%) and liver transplantations (21,064 of 60,002; 35,1%) between 2002 and 2014.

We identified several factors associated with waitlist mortality and/or post-transplant survival. Among those, female gender was associated with an increased risk of waitlist mortality or of becoming too sick for transplantation. Several studies have reported similar results with an increased risk of waitlist mortality among females in the MELD era [13]. One proposed reason is that women have lower muscle mass which results in lower creatinine levels and thus lower MELD scores [14]. Still, models adjusting for glomerular filtration rate (GFR) found a higher waitlist mortality among women [14, 15]. Another possible explanation relates to the smaller physical size of women. As a greater percentage of donors are male, and more livers are allocated to men by size matching [15]. Furthermore, HCV-infected women have higher rates of spontaneous HCV clearance and slower progression to cirrhosis than men [3, 16]. Thus, HCV infected women that become waitlist candidates might have other disadvantageous cofactors that lead to rapid disease progression and increased risk of mortality. Hence, greater attention should be given HCV infected women on the waitlist to optimize pre-transplant care with treatment of risk factors (e.g. diabetes, hypertension, infection). Similarly, our analysis revealed an increased mortality of female transplant recipients, which is in line with earlier reports of much smaller size [17, 18]. This is a unique feature of HCV as no sex differences in post-transplant survival have been reported for transplants due to alcoholism, autoimmune liver disease or NASH [19]. In HCV the worse outcomes of female transplant recipients are probably related to higher rates of acute rejection and a less successful treatment response to interferon treatment of HCV recurrence after liver transplantation [20]. The latter disadvantage, however, is likely to be equalized with the implementation of interferon-free, all-oral direct acting antiviral treatment options in the pre- and post-transplant setting.

In this study, age was associated with waitlist mortality and post-transplant survival. This seems especially relevant since the mean age of the HCV cohort increased more rapidly during the last decade than for any other liver disease [21]. However, it was shown recently that the transplant related survival-benefit was not affected substantially by age since older patients already have a lower survival without transplantation than younger patients [21]. The current study reports an association of increasing donor age with post-transplant survival although the small size of this effect (HR 1.02 per year) makes it a rather weak limiting factor. Accordingly, the effect of donor age on transplant outcome is controversial with some indications that higher donor age is associated with increased post-transplant complications and mortality [22].

In the current study, we report absence of race/ethnicity-related differences in waitlist mortality when adjusted for region and other relevant covariates. A prior study analyzed the impact of ethnicity in the pre- and post-MELD eras demonstrating that an increased waitlist mortality among black patients in the pre-MELD era was equalized once MELD was implemented [23]. Nevertheless, there are indications that minority populations are less likely to be both screened for HCV and referred for liver transplantation [24]. However, the proportion of UNOS/OPTN registrations for HCV among black persons has increased over the last decade, which might be a consequence of steady advances in this field.

The post-transplant survival of HCV patients was significantly associated with recipient race/ethnicity with worse outcomes among black patients. These finding confirms a 2008 UNOS analysis of the whole transplant cohort that found increased mortality among black patients. This association, however, was not observed among non-HCV patients indicating it to be a HCV-related effect [25]. Reasons for this might be lower response rates to interferon among black patients and an accelerated progression of liver fibrosis after HCV recurrence in the transplanted liver [26, 27]. The study observed the best post-transplant survival for patients with Hispanic ethnicity which has been reported previously [28]. The improved treatment possibilities with DAAs should balance therapy related disparities of post-transplant mortality. Nevertheless, persisting general health disparities among racial/ethnic groups in the US might continue to affect differences of transplant outcomes in the future. In addition, in the current study donor race/ethnicity was associated with post-transplant survival with higher hazard ratios associated with Asian and Hispanic donors. Asrani et al. reported similar results in a mixed liver transplant cohort of 10,874 patients, but the results were not significant when adjusted for donor age, height, HBV status and transplant center [29]. Still, there is evidence that a racial/ethnic mismatch between donor and recipient in HCV infected patients is associated with increased risk for graft failure [30]. This might contribute to the observed association in the UNOS HCV cohort, since the absolute number of liver donors of each racial/ethnic group depends on its proportion in the general population and thus donor livers of racial/ethnic minorities are more likely to be transplanted to a recipient of a different group.

The current study reports an association between waitlist mortality and blood type, with the highest risk of waitlist mortality occurring among type 0 patients followed by types A, B and AB. The increased risk of waitlist mortality or becoming too sick to transplant for patients with blood type 0 illustrates how genetic prerequisites can influence allocation probabilities. This finding was reported previously and is probably due to ABO-compatible organ allocation increasing the liver supply for candidates with blood types B and AB, decreasing it for type 0 and A candidates [31, 32]. Conversely, blood type had no association with post-transplant survival indicating that transplantations were not conducted too late among patients with blood type 0. Nevertheless, allocation policies should be refined to balance blood type related disparities in liver allocation.

One of the major risk factors associated with waitlist and post-transplant survival in the current study was presence of diabetes. Additionally, diabetes of the liver donor was associated with reduced post-transplant survival. A recent analysis of UNOS data reported no association between waitlist mortality and presence of diabetes in HCV patients, while an association with post-transplant survival was observed [33]. However, the study used BMI as an indicator of obesity, which is questionable in a cohort of liver disease patients in which there was high percentage of patients with ascites. Patients with diabetes were compared with non-diabetes/non-obese patients making the analysis again dependent on bias-prone BMI. A recent systematic review provided strong evidence of accelerated fibrosis progression and morbidity among HCV patients with diabetes [34]. Thus, close monitoring of diabetes and its complications as well as comprehensive diabetes treatment may reduce pre- and post-transplant mortality of HCV patients. This is especially relevant since the prevalence of diabetes and metabolic syndrome is on the increase in the United States and nonalcoholic steatohepatitis (NASH) itself is a major cause for end stage liver disease [35].

The differences in waitlist mortality and post-transplant survival according to transplant region in the US have been reported and discussed previously [36]. Currently there are proposals to reduce the number of transplant regions to balance geographical disparities in liver allocation [37].

Liver transplantation for HCCs matching Milan criteria is well established and post-transplant outcomes are comparable to those of non-HCC transplantations [38]. Due to the exception policies established with the beginning of the MELD era, patients with HCC have shorter waiting times before transplantation and a lower risk of mortality on the waitlist [39]. In our study, presence of HCC was the strongest predictor of waitlist survival, reflecting the high impact of the exception policies. In 2013, 40% of transplantations in the cohort were performed in patients with HCC. To balance the allocation, with and without exception points, revised allocation regulations were implemented recently in the U.S. ("Share 35 Regional" policy) [40]. Given the notable impact of exception policies on the HCV cohort, these adoptions will have implications for HCV patients on the waitlist in balancing the advantages for patients with HCC. However, the finding that having HCC had no influence on post-transplant survival of HCV patients indicates that the exception policy is elementary for curative cancer treatment by liver transplantation for those patients.

According to the time trend analyses the percentage of HCC patients among the HCV waitlist and transplant cohort tremendously increased during the period of this study. This development is the consequence of several conditions and developments. The aging of the HCV cohort in the United States especially of the group of "baby boomers" has led to an increase of HCV related complications such as development of cirrhosis and HCC. A study of over 5 million US veterans reported that the prevalence of HCV-related cirrhosis increased from 252 to 530 per 100,000 from 2001 to 2013. In the same period HCV-related HCC more than tripled from 8 to 30 per 100,000 [41]. Even in recent years with highly effective DAA regimens available, the numbers of HCC on the waitlist were increasing [12]. Together with the aging HCV cohort the number of individuals that are unaware of their infection and present with advanced stages of liver disease will increase [42]. It is estimated that by 2020, there will be 560,000 individuals who are unaware that they are HCV infected [43]. The unsatisfying SVR rates of historic interferon based treatment regimes failed to counteract these trends. As a consequence, HCV-associated mortality is higher than for any other infectious disease, as reported recently [8]. Further reasons for the increase of patients with HCC among transplant candidates and recipients are probably an improvement of HCC surveillance strategies and diagnostic techniques leading to earlier diagnoses at stages where patients are still eligible for transplantation.

While the current developments in the HCV field already have led to a reduction of the HCV disease burden, several issues for patients with advanced disease remain [10]. Direct acting antiviral agents have tremendously improved SVR rates and also have offered treatment options for patients with advanced liver disease. SVR has been shown to reduce overall HCV-related mortality. In patients with very advanced liver disease (Child-Pugh B/C cirrhosis), prospective studies demonstrating that SVR may preclude liver transplantation in patients with advanced liver disease or prevent death, are currently lacking. Although SVR has been shown to improve biochemical characteristics and portal hypertension in some patients, variables allowing the identification of patients with decompensated liver disease who may benefit from treatment are poorly defined [44]. It is also unclear whether SVR can prevent short-term risk of HCC (recurrence). Initially even some studies have reported a high incidence of HCC and unexpectedly early tumor recurrence among patients successfully treated with DAAs [45, 46]. This finding was not confirmed by larger prospective cohorts [47, 48].

Given these developments, the management of patients with HCV-related end stage liver disease will remain a challenging task. A total of 320,000 deaths, 157,000 cases of HCC, and 203,000 cases of cirrhosis are predicted in the United States for the upcoming 35 years even when the current available highly effective treatment regimens are taken into account [43]. As a consequence, the identified risk factors of waitlist and post-transplant survival of HCV infected patients should receive special attention in patient care and allocation policies. Concerted efforts are necessary to increase diagnosis and treatment rates, optimize care of patients with cirrhosis and HCC and provide care to liver transplant recipients to reduce the overall HCV-related disease burden in the United States.

## Supporting information

S1 Fig Selection process, inclusion and exclusion criteria of the study cohort. Of 137,589 registrations for liver transplantation between 2002 and 2014, 41,157 were included into the study.
(TIF)

S1 Table Demographic and baseline characteristics of the overall study population by time period (N = 41,157). (DOCX)

S2 Table Demographic and baseline characteristics of the HCC subgroup by time period (N = 11,075). (DOCX)
