# Supplementary figures and images for: Liver transplantation for chronic hepatitis C virus infection in the United States 2002–2014: An analysis of the UNOS/OPTN registry
Source: PLoS One. 2017 Oct 31;12(10):e0186898. doi: 10.1371/journal.pone.0186898 (PMC5663425; doi:10.1371/journal.pone.0186898)

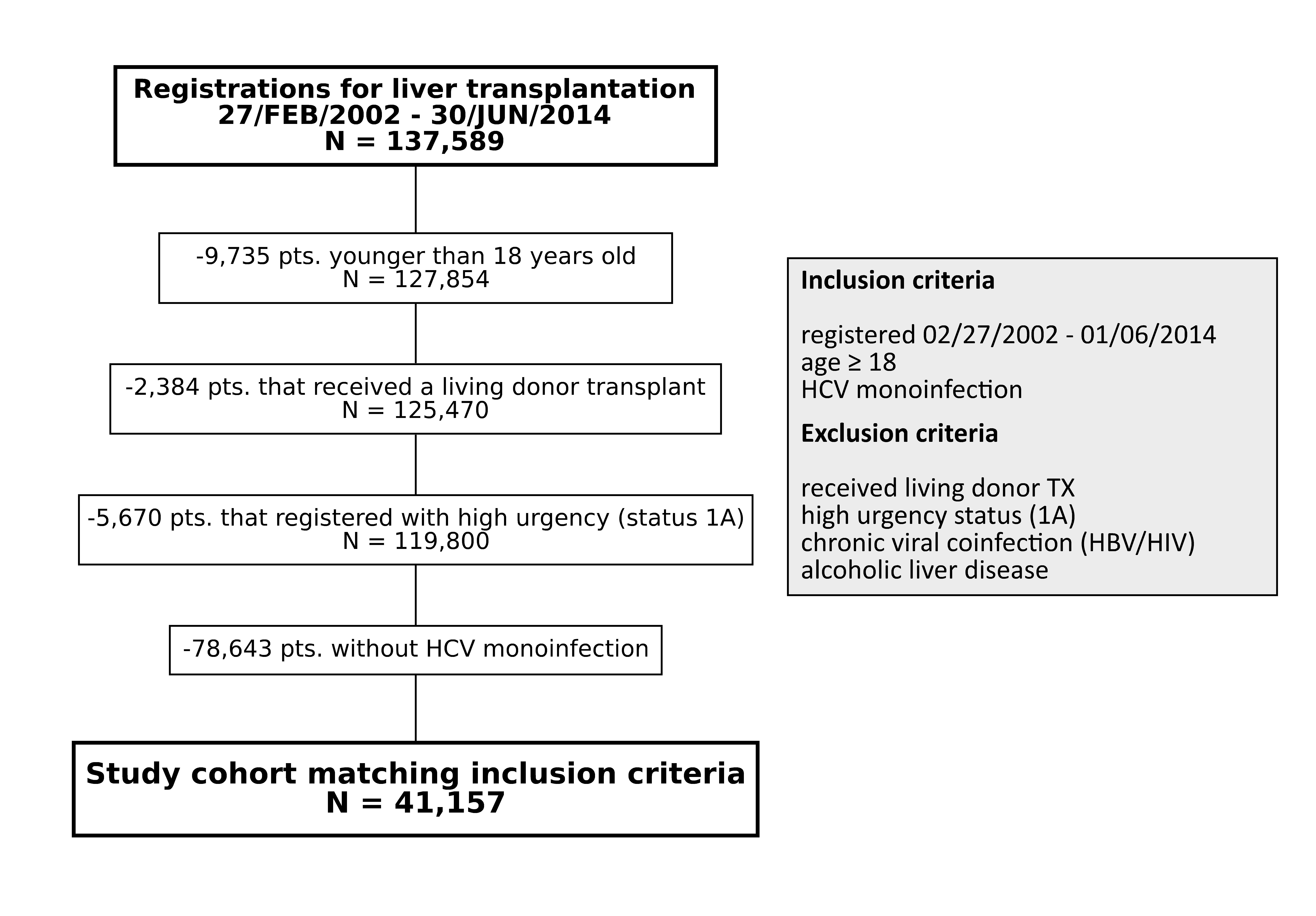

Supplement: S1 Fig — Of 137,589 registrations for liver transplantation between 2002 and 2014, 41,157 were included into the study. (TIF) [file pone.0186898.s001.tif]
