# Supplementary material for: Liver transplantation for chronic hepatitis C virus infection in the United States 2002–2014: An analysis of the UNOS/OPTN registry
Source: PLoS One. 2017 Oct 31;12(10):e0186898. doi: 10.1371/journal.pone.0186898 (PMC5663425; doi:10.1371/journal.pone.0186898)
Supplement: S2 Table — (DOCX) [file pone.0186898.s003.docx]

| **Table S1b: Demographic and baseline characteristics of the HCC subgroup by time period (N = 11,075)** | | | | |
| --- | --- | --- | --- | --- |
|  | 2002-2005 (N= 1,771) | 2006-2009 (N = 3,502) | 2010-2014 (N = 5,802) | p-value^**^ |
| Male sex, N (%) | 1,384 (78.2) | 2,742(78.3) | 4,497(77.5) | 0.6399 |
| Age, mean ± SD |  |  |  |  |
| Male | 53.9 ± 6.8 | 55.9 ± 6.0 | 58.4 ± 5.6 | <0.0001 |
| Female | 56.6 ± 8.1 | 57.8 ± 6.9 | 69.3 ± 6.7 | <0.0001 |
| BMI mean ± SD [kg/m²] |  |  |  |  |
| Male | 28.2 ± 4.6 | 28.5 ± 4.8 | 28.4 ± 4.7 | 0.2102 |
| Female | 27.5 ± 5.1 | 28.6 ± 6.0 | 28.6 ± 5.7 | 0.0069 |
| Ethnicity, N (%) |  |  |  | <0.0001 |
| White | 1,153 (65.1) | 2,309 (65.9) | 3,765 (64.9) |  |
| Black | 170 (9.6) | 428 (12.2) | 803 (13.8) |  |
| Hispanic | 289 (16.3) | 521 (14.9) | 892 (15.4) |  |
| Asian | 139 (7.9) | 197 (5.6) | 273 (4.7) |  |
| Other | 20 (1.1) | 47 (1.3) | 69 (1.2) |  |
| Lab-MELD, N (%) |  |  |  | <0.0001 |
| MELD <10 | 567 (32.0) | 1,172 (33.5) | 2,392 (41.2) |  |
| MELD 10-14 | 774 (43.7) | 1,376 (39.3) | 2,112 (36.4) |  |
| MELD 15-19 | 281 (15.9) | 592 (16.9) | 764 (13.2) |  |
| MELD 20-24 | 89 (5.0) | 192 (5.5) | 295 (5.1) |  |
| MELD 25-29 | 19 (1.1) | 80 (2.3) | 93 (1.6) |  |
| MELD 30-34 | 24 (1.4) | 45 (1.3) | 72 (1.2) |  |
| MELD 35+ | 11 (0.6) | 44 (1.3) | 72 (1.2) |  |
| Missing | 6 (0.3) | 1 (0.0) | 2 (0.0) |  |
| Lab-MELD, mean ± SD | 12.3 ± 5.3 | 12.7 ± 5.9 | 12.0 ± 6.0 | <0.0001 |
| Allocation MELD, N (%) |  |  |  | <0.0001 |
| MELD <10 | 563 (31.8) | 1,164 (33.2) | 2,371 (40.9) |  |
| MELD 10-14 | 770 (43.5) | 1,370 (39.1) | 2,099 (36.2) |  |
| MELD 15-19 | 280 (15.8) | 588 (16.8) | 756 (13.0) |  |
| MELD 20-24 | 88 (5.0) | 190 (5.4) | 293 (5.1) |  |
| MELD 25-29 | 19 (1.1) | 79 (2.3) | 92 (1.6) |  |
| MELD 30-34 | 24 (1.4) | 44 (1.3) | 73 (1.3) |  |
| MELD 35+ | 11 (0.6) | 42 (1.2) | 72 (1.2) |  |
| Temporarily Inactive | 16 (0.9) | 25 (0.7) | 46 (0.8) |  |
| Laboratory values, mean± SD |  |  |  |  |
| Albumin | 3.2 ± 0.6 | 3.1 ±0.7 | 3.2±0.7 | <0.0001 |
| Bilirubin | 2.4 ± 3.5 | 2.6 ± 4.3 | 2.3±4.1 | <0.0001 |
| INR | 1.3 ± 0.3 | 1.4 ± 0.4 | 1.3± 0.4 | <0.0001 |
| Serum creatinine | 1.0 ± 0.8 | 1.0 ± 0.8 | 1.0± 0.9 | <0.0001 |
| ABO blood type, N (%) |  |  |  | 0.3556 |
| 0 | 812 (45.9) | 1,646 (47.0) | 2,636 (45.4) |  |
| A | 667 (37.7) | 1,258 (35.9) | 2,193 (37.8) |  |
| AB | 75 (4.2) | 154 (4.4) | 217 (3.7) |  |
| B | 217 (12.3) | 444 (12.7) | 756 (13.0) |  |
| Encephalopathy, N (%) |  |  |  | <0.0001 |
| Absent | 938 (53.9) | 1,991 (56.9) | 3,582 (61.7) |  |
| Present | 825 (46.6) | 1,510 (43.1) | 2,218 (38.2) |  |
| Unknown | 8 (0.45) | 1 (0.0) | 2 (0.0) |  |
| Ascites, N (%) |  |  |  | <0.0001 |
| Absent | 710 (40.1) | 1,510 (43.1) | 2,964 (51.1) |  |
| Present | 1,053 (59.5) | 1,991 (56.9) | 2,836 (48.9) |  |
| Unknown | 8 (0.5) | 1 (0.0) | 2 (0.0) |  |
| Diabetes, N (%) |  |  |  | <0.0001 |
| No | 1,357 (76.6) | 2,627 (75.0) | 4,339 (74.8) |  |
| Yes | 385 (21.7) | 837 (23.9) | 1,451 (25.0) |  |
| Unknown | 29 (1.6) | 38 (1.1) | 12 (0.2) |  |
| Region, N (%) |  |  |  | <0.0001 |
| 1 | 105 (5.9) | 157 (4.5) | 351 (6.1) |  |
| 2 | 181 (10.2) | 417 (11.9) | 843 (14.5) |  |
| 3 | 240 (13.6) | 423 (12.1) | 575 (9.9) |  |
| 4 | 183 (10.3) | 387 (11.1) | 623 (10.7) |  |
| 5 | 416 (23.5) | 756 (21.6) | 1,148 (19.8) |  |
| 6 | 97 (5.5) | 149 (4.3) | 218 (3.8) |  |
| 7 | 92 (5.2) | 176 (5.0) | 309 (5.3) |  |
| 8 | 92 (5.2) | 222 (6.3) | 335 (5.8) |  |
| 9 | 154 (8.7) | 346 (9.9) | 547 (9.4) |  |
| 10 | 126 (7.1) | 234 (6.7) | 387 (6.7) |  |
| 11 | 85 (4.8) | 235 (6.7) | 466 (8.0) |  |

*States and Areas in Regions of OPTN: 1 Connecticut, Maine, Massachusetts, New Hampshire, Rhode Island, Vermont; 2 Delaware, District of Columbia, Maryland, New Jersey, Pennsylvania, Northern Virginia, West Virginia; 3 Alabama, Arkansas, Florida, Georgia, Louisiana, Mississippi, Puerto Rico 4 Oklahoma, Texas; 5 Arizona, California, Nevada, New Mexico, Utah; 6 Alaska, Hawaii, Idaho, Montana, Oregon, Washington; 7 Illinois, Minnesota, North Dakota, South Dakota, Wisconsin; 8 Colorado, Iowa, Kansas, Missouri, Nebraska, Wyoming; 9 New York; 10 Indiana, Michigan, Ohio; 11 Kentucky, North Carolina, South Carolina, Tennessee, Virginia.

^**^ P-values for joint test of differences among the time period specific means for Age, BMI, Lab-MELD, Laboratory values were computed using unadjusted linear regression and among the time period specific proportions for Ethnicity, Lab MELD, Allocation MELD, HCV related HCC, ABO Blood types, Encephalopathy, Ascites, Diabetes, and Region were computed using Chi-square tests.
